# New York College of Veterinary Surgeons

**Published:** 1896-05

**Authors:** 


### NEW YORK COLLEGE OF VETERINARY SURGEONS.

THIS year an innovation was inaugurated, and the graduating class, numbering twenty-nine, were summoned to the college hall at 7 P.M. on April 1st, and, after suitable opening remarks, Prof. H. M. Biggs, President of the Board of Trustees, gave to each graduate the oath of Hippocrates, and then the whole class, alumni, Board of Trustees, and several visitors were invited to the Hotel Marlborough, where was spread one of the most sumptuous banquets ever enjoyed. The table was arranged to seat sixty, and was beautifully decorated with flowers and candelabra. Dean H. D. Gill sat at the head of the table, and at his right Prof. J. R. Huddleston, who discharged the duties of toast-master in a most felicitious manner, adding much to the pleasure of the occasion. Around the head of the table were seated members of the Board of Trustees, Faculty, and President W. Horace Hoskins of the United States Veterinary Medical Association.

The response to the various toasts, as well as the class oration by Dr. C. R. Scattergood, of the graduating class, elicited much interest, and were of a character tending to enlighten all present as well as to convey more forcibly to the students a keener appreciation of the great responsibilities resting upon them. The class orator gave many interesting statistics, which were strong points that the future of the veterinarian was never brighter and that the scope of his work was just beginning to be appreciated and realized. The vein of humor pervading the introduction by the toast-master of those who responded to the various toasts was not the least of a very large part of the enjoyment of the occasion, and was in keeping with the suggestive and artistic arrangement of the program and menu card so tastefully prepared for the occasion, and which will ever remain in the hands of the possessors as a pleasant memento of the occasion. The absence of Prof. Huidekoper, from whose sick-bed a bulletin of health was received and read, exhibiting so keenly the sanguine, jovial character of the man, made more regretful the many expressions of sympathy and sorrow at his enforced absence. We append a list of the graduates with their addresses:

Louis Abel, Brooklyn, N. Y.; Joseph Cohen, New York; John Hambleton Dedrich, Schenectady, N. Y.; George Clark De Witt, Oak Hill, N. Y.; James Thomas Glennon, Newark, N. J.; William Patrick Hanifen, New York; George Ferdinand Harpel, Jersey City, N. J.; Milton Daniel Harper, Philadelphia, Pa.; John Francis Harrison, Stanford, Conn.; George Edgar Harder, Pittsfield, Mass.; J. Homer Huff, Paterson, N. J.; Augustus F. Johnson, New York; Samuel Henry Johnson, Philadelphia, Pa.; Eckford S. E. Littlefield, Brooklyn, N. Y.; James Francis Laden, New Haven, Conn.; James Brown McNeil, Albany, N. Y.; Charles Emerson May, Avon, Mass.; Daniel Girard O'Connell, Brooklyn, N. Y.; Nathan Peyser, New York; Thomas Michael Quinn, Astoria, L. I.; William Riley Rhinehart, Inwood, L. I.; Robert Guernsey Rice, Rome, Pa.; Joseph Schnurmacher, New York; Charles R. Scattergood, Brooklyn, N. Y.; Major Schofield, Brooklyn, N. Y.; Charles R. Utz, Freeport, L. I.; John Tjaden, New York; Max Wendell, Middle Village, L. I.; Walter H. Yingst, Harrisburg, Pa.

